# Serum metabolite profile associated with incident type 2 diabetes in Koreans: findings from the Korean Genome and Epidemiology Study

**DOI:** 10.1038/s41598-018-26320-9

**Published:** 2018-05-29

**Authors:** Soo Jin Yang, So-Young Kwak, Garam Jo, Tae-Jin Song, Min-Jeong Shin

**Affiliations:** 1 Department of Food and Nutrition, Seoul Women’s University, Seoul, 01797 Republic of Korea; 2 Department of Public Health Sciences, BK21PLUS Program in Embodiment: Health-Society Interaction, Graduate School, Korea University, Seoul, 02841 Republic of Korea; 3 Department of Neurology, Ewha Womans University School of Medicine, Seoul, 07985 Republic of Korea

## Abstract

The identification of metabolic alterations in type 2 diabetes (T2D) is useful for elucidating the pathophysiology of the disease and in classifying high-risk individuals. In this study, we prospectively examined the associations between serum metabolites and T2D risk in a Korean community-based cohort (the Ansan–Ansung cohort). Data were obtained from 1,939 participants with available metabolic profiles and without diabetes, cardiovascular disease, or cancer at baseline. The acylcarnitine, amino acid, amine, and phospholipid levels in fasting serum samples were analyzed by targeted metabolomics. During the 8-year follow-up period, we identified 282 cases of incident T2D. Of all metabolites measured, 22 were significantly associated with T2D risk. Specifically, serum levels of alanine, arginine, isoleucine, proline, tyrosine, valine, hexose and five phosphatidylcholine diacyls were positively associated with T2D risk, whereas lyso-phosphatidylcholine acyl C17:0 and C18:2 and other glycerophospholipids were negatively associated with T2D risk. The associated metabolites were further correlated with T2D-relevant risk factors such as insulin resistance and triglyceride indices. In addition, a healthier diet (as measured by the modified recommended food score) was independently associated with T2D risk. Alterations of metabolites such as amino acids and choline-containing phospholipids appear to be associated with T2D risk in Korean adults.

## Introduction

Type 2 diabetes (T2D) is an increasingly prevalent metabolic disorder that causes serious micro- and macrovascular complications^1^. In Korea, the prevalence of diabetes and its accompanying cardiovascular disease burden have continuously increased as dietary habits become more westernized^2,3^. Therefore, identifying novel risk factors of T2D along with well-known factors such as insulin resistance or insufficient insulin secretion is important because proper screening can lower or delay T2D development.

Unlike genes or proteins, metabolites are biomarkers of the biochemical activity and are closely related to clinical phenotypes^4^. They can also serve as a good indicator of enzyme activity resulting from biological process of transcription and translation, allowing the monitoring of systemic changes in biological systems. Accordingly, metabolite analysis provides a functional readout of the physiological state of phenotypes, revealing previously undetected biological mechanisms that underlie diseases and metabolic pathways^5^. Therefore, through metabolic profiling, we can identify individuals or populations at a high risk for developing T2D and seek methods of controlling the T2D occurrence.

Several studies have confirmed the association between metabolites and new-onset T2D. In a recent meta-analysis of eight prospective studies, several blood amino acids (leucine, valine, tyrosine, and phenylalanine) were positively associated with T2D risk, whereas others (glycine and glutamine) were negatively associated with T2D risk^6^. The European Prospective Investigation into Cancer and Nutrition (EPIC)-Potsdam cohort identified serum metabolites such as hexoses, phenylalanine, and diacyl-phosphatidylcholines (C32:1, C36:1, C38:3 and C40:5) to be potential predictors of incident T2D^7^. Another prospective study in the Framingham Offspring Study cohort with a 12-year follow-up revealed that amino acids, namely isoleucine, leucine, valine, tyrosine, and phenylalanine, either singly or in combination increased the odds ratio for developing future diabetes^8^. Through similar approaches, a nested case–control design by the Framingham Heart Study cohort suggested that metabolite 2-aminoadipic acid is a strong marker of T2D onset^9^.

Although blood metabolite profiling of T2D pathogenesis has accumulated much knowledge in the western population^7–9^, efforts to identify predictive metabolites of new-onset T2D in the Asian population are limited. A recent metabolic analysis reported a distinctive metabolic signature, including palmitoylcarnitine, for incident T2D in the Chinese population^10^. Untargeted metabolomics analysis suggested a combination of six metabolites including proline, glycerol, aminomalonic acid, lysophosphatidylinositol (LPI) (16:1), 3-carboxy-4-methyl-5-propyl-2-furanpropionic acid and urea to have potentials to predict the development of T2D in Chinese population with high risk of T2D^11^. Another targeted metabolomics analysis identified 37 metabolites including LPI (16:1) and dihomo-γ-linolenic acid that are associated with incident T2D in a subset of the Singapore Chinese Health Study cohort^12^. Previous studies have identified several T2D-associated metabolites in Korean populations, but these studies were not prospective^13,14^. Therefore, the present study aims to identify the metabolites related to incident T2D in large populations by studying a prospective cohort in Korea. Whether the dietary pattern is related to the observed association between metabolites and incident T2D is also discussed.

## Subjects and Methods

### Study participants

**Figure 1:**
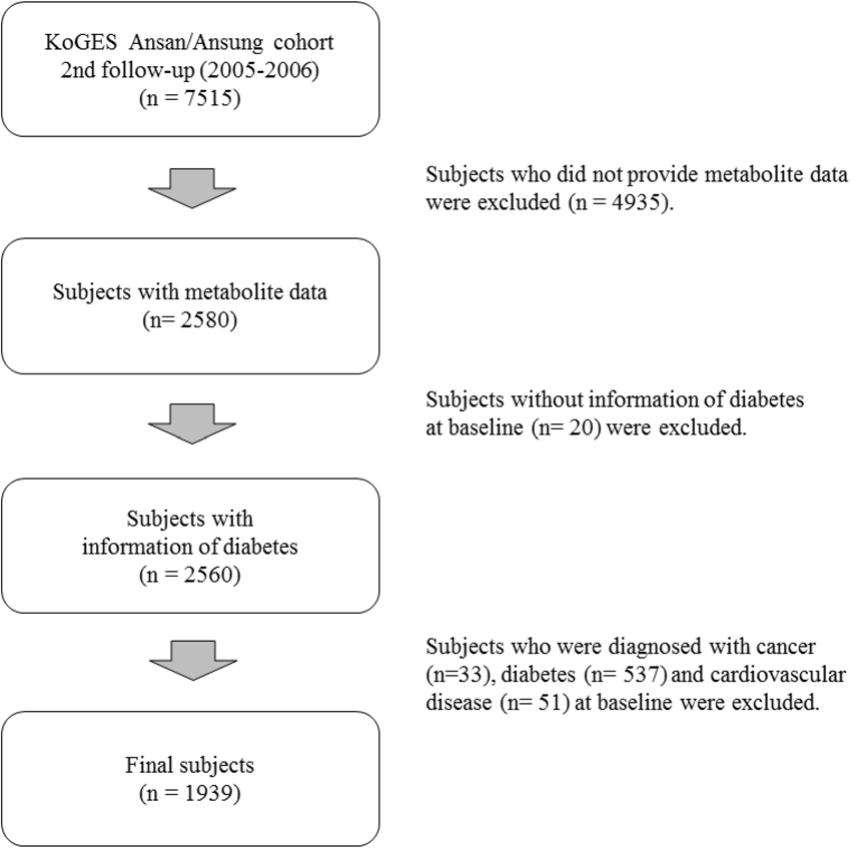
Flow chart of study population.

The study population was assembled through the Korean Genome and Epidemiology Study (KoGES) (the Ansan–Ansung study). The procedure and design of the KoGES-Ansan and Ansung cohorts are described elsewhere^15,16^. Briefly, 10,030 individuals aged 40–69 years living in the Ansan (urban) and Ansung (rural) districts were recruited as the baseline in 2001–2002. The aim was to construct a genomic and epidemiologic database for examining the genetic and environmental effects on disease prevalence in Korean. The participants attended questionnaire-based interviews in a community clinic, where they were questioned on their sociodemographic information, lifestyle, health condition, and medical history. They were also subjected to anthropometric measurements and clinical examination, and biennial follow-up examinations. Our dataset was obtained from the second follow-up (2005–2006) of the KoGES study. Among the 7,515 participants, we selected 2,580 participants whose metabolite information was available. After excluding subjects with no data on diabetes (n = 20), preexisting cancer (n = 33), diabetes (n = 537), and cardiovascular diseases (n = 51) at the time of enrolment in the study, 1,939 subjects were recruited. Patients with cancer, diabetes, and cardiovascular diseases were excluded because their medical treatments could alter their metabolic profiles (Fig. 1). All study participants gave their informed consent. The study protocol was approved by the Institutional Review Board of the Korea Centers for Disease Control and Prevention^17,18^, and by the Institute Review Board at the Korea University (KU-IRB-16-EX-272-A-1). All experiments were performed in accordance with relevant guidelines and regulations.

### General data and anthropometric and biochemical measurements

The demographic and behavioral information of the participants (i.e., information on age, sex, physical activity, cigarette smoking, and alcohol consumption) was obtained from survey questionnaires administered by trained interviewers. The income level (monthly household income) was divided into lowest (<1 million Korean won), lower-middle (100–199 thousand Korean won), upper-middle (200–399 thousand Korean won), and highest (≥4 million Korean won). Here 1,000 Korean won approximately corresponds to 0.9 US dollars. Smoking and drinking statuses were classified into three categories: never, former, and current. Participants were asked how long they had participated in five types of activities (sedentary, very light, light, moderate, and intense physical activities). The total metabolic equivalents (METs) were calculated by summing the METs during each type of activity (1.0 for sedentary, 1.5 for very light, 2.4 for light, 5.0 for moderate, and 7.5 for intense)^19^. Height and body weight were measured to the nearest 0.1 cm and 0.1 kg, respectively, while wearing light clothes without shoes. The body mass index (BMI; kg/m^2^) was calculated as the weight divided by the squared height. Blood pressure was repeatedly measured by a trained technician using a mercury sphygmomanometer. The participant assumed a sitting position and the blood pressure was read from the left and right arms, with a 5-minute rest between the two readings. The measurements were recorded to the nearest 2 mmHg and averaged to obtain the systolic and diastolic blood pressures. Hypertension was defined as a systolic blood pressure of  ≥140 mmHg or a diastolic blood pressure of ≥90 mmHg, a doctor-diagnosis of hypertension by the participants, or the taking of anti-hypertensive medication. To assay their fasting plasma glucose (FPG; mg/dL), fasting plasma insulin (FPI; μIU/mL) and triglycerides (TG; mg/dL), participants were required to fast for at least 8 h before providing a blood sample. The assays were performed by an automatic analyzer (ADVIA 1650 and 1680, Siemens, Tarrytown, NY, USA). Glycosylated hemoglobin (HbA_1_c) was measured by high-performance liquid chromatography (Variant II, Bio-Rad, Hercules, CA, USA). Homeostasis model assessment of insulin resistance (HOMA-IR) was calculated as follows^20^: HOMA-IR = [FPI (μIU/mL) × FPG (mg/dL)]/450.

### Metabolite measurements

The serum metabolites in the 2,580 subjects were quantitatively analyzed by a targeted metabolomics approach using the AbsoluteIDQTM p180 kit (BIOCRATES Life Sciences, Innsbruck, Austria), which contains 40 acylcarnitines, 21 amino acids, 19 biogenic amines, 1 hexose, 90 glycerophospholipids, and 15 sphingolipids. The experimental procedure and sample measurements in the present study are described in detail elsewhere^13,14^. Briefly, 10 µL of serum was aliquoted on a 96-well plate with a filter and then centrifuged for 2 min at 100 × g. After extraction of metabolites, the extracts were analyzed by flow injection analysis/tandem mass spectrometry (FIA–MS/MS) for the analysis of acylcarnitines, hexose, glycerophospholipids and sphingolipids in both positive (acylcarnitines, glycerophospholipids, and sphingolipids) and negative (hexose) ion mode. Amino acids and biogenic amines were quantified by using liquid chromatography/tandem mass spectrometry (LC–MS/MS) in positive ion mode. Internal standards labeled with stable isotopes, such as ^13^C or ^15^N, were used as reference for the identification and quantification of all metabolites provided with the AbsoluteIDQTM p180 kit. The metabolite concentration measurement in uM units was automatically carried out with the MetVal^TM^ software package (BIOCRATES Life Sciences AG, Innsbruck, Austria), and quality assurance was performed for the quality assessment using calibration standards and QC samples included on each plate as well as reference standards as normal human pooled serum. Data quality of each metabolite was checked based on the following criteria: (1) the coefficient of variance for each metabolite in the reference standards <25%, (2) half of the analyzed metabolite concentrations in the reference standards > limit of detection, and (3) half of the analyzed metabolite concentrations in the experimental samples > limit of detection. After excluding 51 metabolites that failed the quality criteria, we finally used 135 metabolites (13 acylcarnitines, 21 amino acids, 10 biogenic amines, 1 hexose, 78 glycerophospholipids, and 12 sphingolipids) for the present study.

### Dietary assessment

Dietary assessments were collected through a validated semi-quantitative food frequency questionnaire (FFQ), which records the consumption frequencies and portion sizes of 103 food items^21^ (see the previous study for details). The frequency of dietary consumption was divided into nine categories: never or seldom, once a month, 2-3 times a week, 1-2 times a week, 3-4 times a week, 5-6 times a week, once daily, twice daily, or more than three times daily. For the statistical analyses, the daily consumption frequencies were converted into weekly consumption frequencies. The serving size of each food item was categorized as small, medium, or large. Participants were also asked about the daily frequency of their meals: one meal a day, two meals a day, three meals a day, more than four meals a day, or irregular. From this information, we calculated the recommended food score (RFS), and hence measured the diet quality in the study population.

### Recommended food score

RFS is a dietary score based on the consumption frequency of food items that are emphasized in the current dietary guidelines for Americans, following the methods of Kant *et al*.^22^. In this study, we modified RFS for consistency with the current dietary guidelines for Koreans^23^. Our RFS included 54 previously validated food items with the following slight modifications^24,25^; whole grain (two items; barley, steamed rice with barley), legumes (four items; steamed rice with cong, tofu, miso soup or soybean paste, soy milk), vegetables (20 items; green chilies, pepper leaves, spinach, lettuce, wild sesame leaves, chives, green vegetables, white radish, bellflower, onion, Chinese cabbages (excluding kimchi), cucumbers, soybean sprouts, carrots, pumpkin, young pumpkin, brackens, vegetable wrappings, oyster mushrooms, other mushrooms), fruits including juice (12 items; persimmons, tangerines, melons, bananas, pears, apples, oranges, watermelons, peaches, strawberries, grapes, tomatoes), fish (nine items; sashimi, hairtail, eel, yellow corbina, mackerel, mackerel pike, anchovy, tuna, fish cakes), seaweed (two items; laver, kelp), dairy products (three items; milk, yogurt, cheese), nuts (one item; peanuts or almonds or pine nuts) and tea (one item; green tea). One point was awarded for each food item consumed at least once a week. Participants were assigned an additional score of 1 if they ate three meals daily. Missing dietary variables including daily frequency of meals were imputed by fully conditional specification approach^26^. RFS was computed by summing the points for each of the 54 recommended food items listed in the FFQ, plus the daily-frequency-of-meals score. Therefore, the total score can range from 0 to 55, with a higher score indicating better diet quality.

### Follow-up and incident T2D

Participants were followed up at two-yearly intervals. At each follow-up examination, the subjects were administered the questionnaire, FPG, and a 2-h oral glucose tolerance test. New-onset T2D was defined by at least one of the following criteria: self-reported diagnosed diabetes, treatment with a hypoglycemic medication, FPG levels of ≥126 mg/dL, or plasma glucose levels of ≥200 mg/dL after the oral glucose tolerance test^18^.

### Statistical analysis

All continuous and categorical values were expressed as mean ± standard error and as number of counts, respectively. Differences among the modified RFS quartiles were determined by one-way variance and a general linear model with a Bonferroni’s multiple comparisons test, in which the possible confounding factors were sex, age, energy intake, metabolic equivalent, smoking status, household income, and educational level. The level of each metabolite was log-transformed and normalized to z scores with a mean of 0 and a standard deviation of 1. Some variables outside of the normal distribution were also log-transformed. Whether a metabolite was associated with new-onset T2D was determined by a multivariable Cox regression analysis. The association between the modified RFS and T2D risk was tested similarly. Finally, the selected metabolites (phosphatidylcholine diacyl [PC aa] 32:1 and hydroxysphingomyelin [SM(OH)] 22:2) and the dietary quality score were adjusted in the model, both alone and in combination. The hazard ratios (HRs) were obtained by a Cox proportional hazards regression model, adjusting for sex, age, BMI, educational level, household income, smoking status, drinking status, METs, total energy, consumptions of coffee, red meat and whole grain, and history of hypertension. The p value was corrected for the false discovery rate (FDR) by the Benjamini–Hochberg method (q < 0.05). The cross-sectional association between the metabolites and the modified RFS was evaluated by a multiple linear regression model, adjusting for age, sex, energy intake, METs, smoking status, drinking status, household income and educational level. All analyses were performed using Stata SE 13.0 (Stata Corp, College Station, TX, USA) with a two-sided p value of <0.05 signifying statistical significance.

### Data availability

All data generated or analyzed during this study are included in this published article and its Supplementary Information files.

## Results

The average age of the 1,939 participants was 56.6 ± 0.2 years, and 892 (46.0%) were males. Table 1 displays the baseline characteristics of the study participants in the quartiles of modified RFS. The subjects in the highest modified RFS quartile were significantly younger, had a higher education and household income, smoked less and exhibited lower metabolic equivalent than subjects in the other groups. All nutrient intakes, including the percentage of macronutrients, significantly differed among the quartiles of modified RFS (Supplementary Table S1). After adjusting for confounders, high-density lipoprotein-cholesterol was significantly higher in the highest quartile of RFS.

**Table 1:** Baseline characteristics of the study population.

	Total (n = 1939)	Recommended food score (RFS)				p-value^†^
		Q1 (n = 465)	Q2 (n = 486)	Q3 (n = 480)	Q4 (n = 508)	
Score, mean/median (range)	19.6/20 (0–48)	7.6/8 (0–12)	16.1/16 (13–19)	22.5/22 (20–25)	31.2/30 (26–48)	—
Age, years	56.6 ± 0.2	59.9 ± 0.4^a^	58.3 ± 0.4^b^	55.2 ± 0.4^c^	53.2 ± 0.3^d^	<0.001
**Sex %, (n)**						
Male	46.0 (892)	46.7 (217)	45.5 (221)	49.6 (238)	42.5 (216)	0.165
Female	54.0 (1,047)	53.3 (248)	54.5 (265)	50.4 (242)	57.5 (292)	
Body mass index, kg/m^2^	24.3 ± 0.1	24.2 ± 0.2	24.2 ± 0.1	24.3 ± 0.1	24.5 ± 0.1	0.349
**Education %, (n)**						
Elementary	40.9 (792)	62.3 (289)	50.0 (243)	31.6 (151)	21.5 (109)	<0.001
Middle school	20.3 (392)	17.7 (82)	21.2 (103)	20.7 (99)	21.3 (108)	
High school	27.7 (535)	15.3 (71)	21.6 (105)	33.9 (162)	38.9 (197)	
University	11.2 (216)	4.7 (22)	7.2 (35)	13.8 (66)	18.3 (93)	
**Income status %, (n)**						
Lowest	37.4 (718)	59.0 (269)	44.5 (215)	29.3 (140)	18.7 (94)	<0.001
Lower middle	22.9 (439)	22.8 (104)	25.3 (122)	20.5 (98)	22.9 (115)	
Upper middle	27.7 (531)	14.7 (67)	22.8 (110)	36.2 (173)	36.0 (181)	
Highest	12.1 (232)	3.5 (16)	7.5 (36)	14.0 (67)	22.5 (113)	
**Smoking status %, (n)**						
Never	63.1 (1,222)	60.0 (279)	64.2 (312)	60.8 (292)	66.9 (339)	<0.001
Former	16.7 (323)	13.8 (64)	14.8 (72)	20.4 (98)	17.6 (89)	
Current	20.3 (393)	26.2 (122)	21.0 (102)	18.9 (90)	15.6 (79)	
**Drinking status %, (n)**						
Never	48.3 (935)	52.0 (242)	49.8 (242)	45.2 (217)	46.2 (234)	0.358
Former	4.3 (84)	4.7 (22)	3.9 (19)	4.4 (21)	4.3 (22)	
Current	47.4 (919)	43.2 (201)	46.3 (225)	50.4 (242)	49.5 (251)	
Metabolic equivalent (h)	53.5 ± 0.4	58.2 ± 0.8^a^	54.9 ± 0.8^b^	50.0 ± 0.7^c^	51.1 ± 0.7^c^	<0.001
Energy intake, kcal	1775.2 ± 13.6	1449.5 ± 19.6^a^	1665.2 ± 22.2^b^	1825.7 ± 22.8^c^	2127.0 ± 31.5^d^	<0.001
Hypertension %, (n)	31.6 (613)	37.2 (173)	34.4 (167)	30.2 (145)	25.2 (128)	<0.001

Values are expressed as means ± standard error for continuous variables and percentages and numbers counts for categorical variables. ^†^Statistical differences were determined using chi-square test for categorical variables and one-way analysis of variance (ANOVA) for continuous variables with Bonferroni’s multiple correction (p < 0.05).

### Prospective associations between metabolites and T2D risk

**Figure 2:**
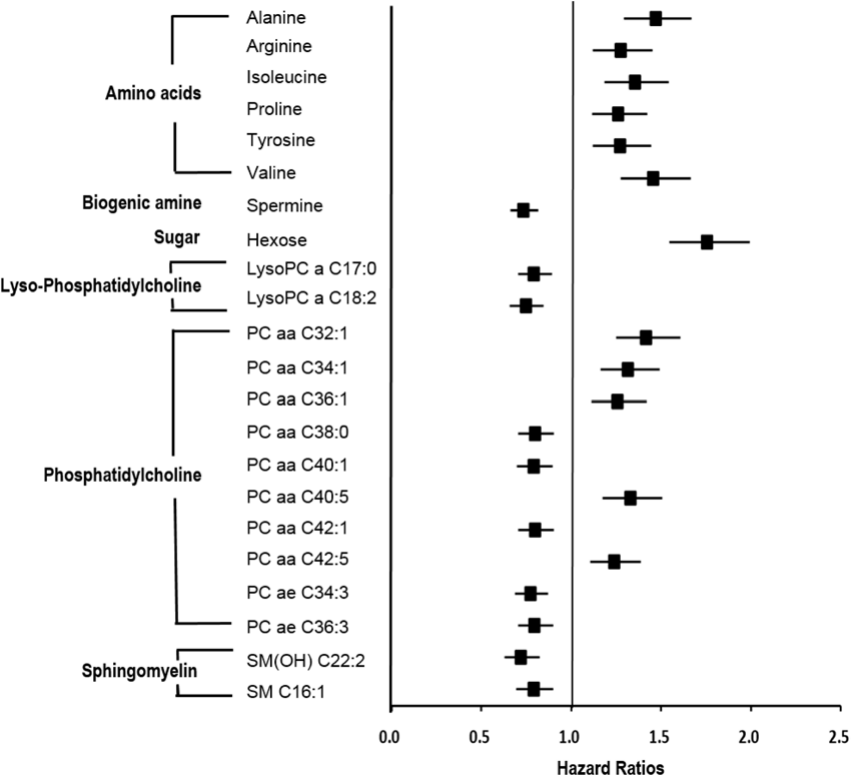
Metabolites being associated with future Type 2 diabetes mellitus risk. Hazard ratios were obtained with cox proportional hazards regression model adjusting for sex, age, energy intake, body mass index, metabolic equivalent, smoking status, drinking status, household income, and education level, consumption of coffee, red meat, and whole grain, and history of hypertension. LysoPC a: lyso phosphatidylcholine acyl; PC aa: phosphatidylcholine diacyl; PC ae: phosphatidylcholine acyl-alkyl; SM(OH): hydroxysphingomyelin; SM: sphingomyelin.

During the 8-year follow-up period, 282 incidents cases of new-onset T2D were identified. Figure 2 presents the metabolites prospectively associated with T2D risk after adjusting for covariates, with a strict correction for multiple comparisons. Twenty-two of the 135 metabolites were significantly related to T2D risk (with FDR-corrected p value of <0.05, Fig. 2 and Supplementary Tables S2). Levels of alanine [HR, 1.47; confidence interval (CI), 1.29–1.67], arginine (HR, 1.27; 95% CI, 1.12–1.45), isoleucine (HR, 1.35; 95% CI, 1.19–1.54), proline (HR, 1.26; 95% CI, 1.12–1.42), tyrosine (HR, 1.27; 95% CI, 1.12–1.45), and valine (HR, 1.46; 95% CI, 1.27–1.66) were positively associated with new-onset T2D. Among the biogenic amines, only spermine was negatively correlated with new-onset T2D (HR, 0.73; 95% CI, 0.66–0.82). Hexose sugar was independently related to new-onset T2D (HR, 1.76; 95% CI, 1.55–1.99). Among the choline-containing phospholipid compounds, lyso-phosphatidylcholine acyls (LysoPC a) C17:0 (HR, 0.79; 95% CI, 0.70–0.89), C18:2 (HR, 0.75; 95% CI, 0.66–0.85), PC aa C38:0 (HR, 0.80; 95% CI, 0.71–0.90), C40:1 (HR, 0.79; 95% CI, 0.70–0.90), and C42:1 (HR, 0.80; 95% CI, 0.71–0.90), along with phosphatidylcholine acyl-alkyls (PC ae) C34:3 (HR, 0.77; 95% CI, 0.69–0.87) and C36:3 (HR, 0.80; 95% CI, 0.70–0.90), were negatively related to new-onset T2D. In contrast, PC aa C32:1 (HR, 1.42; 95% CI, 1.25–1.61), C34:1 (HR, 1.32; 95% CI, 1.16–1.49), C36:1 (HR, 1.26; 95% CI, 1.11–1.42), C40:5 (HR, 1.33; 95% CI, 1.17–1.51), and C42:5 (HR, 1.24; 95% CI, 1.11–1.39) were positively associated with new-onset T2D. Two sphingomyelins (SM), SM(OH) C22:2 (HR, 0.72; 95% CI, 0.63–0.82) and SM C16:1 (HR, 0.79; 95% CI, 0.69–0.90), were negatively correlated with new-onset T2D (Fig. 2).

### Association between selected metabolites and biomarkers for T2D

**Figure 3:**
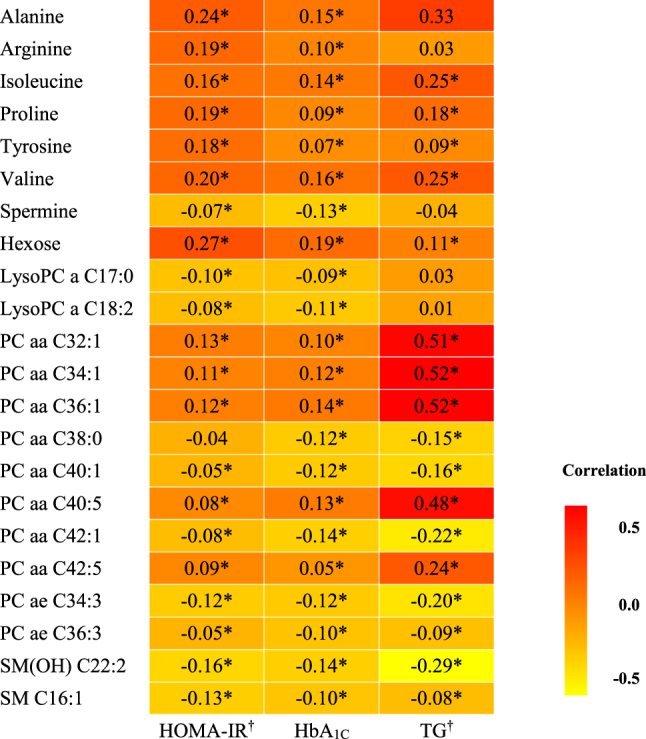
Correlation analysis plot of metabolites and biomarkers. *Correlation coefficients were obtained with partial correlation analysis adjusting for sex, age, energy intake, body mass index, metabolic equivalent, smoking status, drinking status, household income, and education level (*p* < 0.05). ^†^The value of biomarkers used in these analyses were log-transformed. LysoPC a: lyso phosphatidylcholine acyl; PC aa: phosphatidylcholine diacyl; PC ae: phosphatidylcholine acyl-alkyl; SM(OH): hydroxysphingomyelin; SM: sphingomyelin.

Figure 3 illustrates the correlations between the metabolites associated with new-onset T2D risk and the established biomarkers for diabetes (HOMA-IR, HbA_1C_ and TG). The metabolites associated with new-onset T2D were also significantly correlated with the diabetes indicators. The exceptions were PC aa C38:0 for HOMA-IR, and alanine, arginine, spermine, LysoPC a C17:0 and C18:2 for TG (Fig. 3).

### Associations among dietary score, selected metabolites, and incident T2D

Given that 22 metabolites were identified as predictors for incident T2D, we further tested the associations among habitual dietary quality, metabolites, and T2D incidence in this study population. As shown in Table 2, a healthier dietary pattern (as quantified by the modified RFS) was significantly negatively associated with T2D incidence (HR: 0.80 CI: 0.70–0.90) after adjusting for covariates. The cross-sectional associations between dietary score and the 22 selected metabolites are shown in Supplementary Table S3. After multiple linear regression analysis and covariate adjustment, two metabolites that were significantly associated with T2D incidence, namely, PC aa C32:1 and SM(OH) C22:2 (beta coefficients −0.28 and 0.26, respectively), were significantly associated with healthier dietary pattern (FDR-corrected p value of <0.05). We further tested whether the metabolite levels exert a mediating effect in the observed association between modified RFS and T2D incidence. The results showed that both modified RFS and metabolites significantly contribute to future T2D risk, although the magnitudes of the associations were more or less attenuated (Table 2).

**Table 2:** Risk of type 2 diabetes according to metabolites and diet quality score.

	PC aa C32:1		SM(OH) C22:2		Modified RFS	
	HR	p-value	HR	p-value	HR	p-value
Model 1	1.469	<0.001	0.714	<0.001	—	—
Model 2	—	—	—	—	0.795	<0.001
**Model 3**						
PC aa C32:1	1.428	<0.001	—	—	0.837	0.006
SM(OH) C22:2	—	—	0.734	<0.001	0.826	0.003

Hazard Ratios were obtained with cox proportional hazards regression model according to the model adjusted. Basic model: age, sex, area, metabolic equivalent, smoking status, drinking status, household income, education level, and prevalence of hypertension adjusted (data not shown). Model 1: basic model + metabolites adjusted; Model 2: basic model + modified RFS adjusted; Model 3: basic model + metabolites + modified RFS adjusted. The values of metabolites used in this analysis were log-transformed and normalized using z score.

## Discussion

In the present study, various biological pathways, including amino acid and choline-containing phospholipid metabolisms, were altered in the incident T2D group of our community-based cohort sample. Recent Korean reports have implicated seven metabolites (hexose, valine, and five PC aas) in obesity and T2D. These metabolites are related to the fat mass and obesity-associated (FTO) genotype^13^. Altered metabolites associated with genetic loci were also identified in Korean T2D subjects^14^. Extending these previous analyses, which investigated only the cross-sectional association between metabolites and T2D, this study sought the metabolic profiles associated with incident T2D over an 8-year follow-up period.

### Amino acids

Previous studies have identified the branched-chain amino acids (BCAAs) as among the most consistent and important metabolites in dysregulation of the peripheral glucose metabolism. BCAAs and aromatic amino acids have been implicated in insulin resistance^27^, obesity^28^, diabetes risk^8,29,30^ and coronary artery disease^31^. Similarly, increased isoleucine, valine, and tyrosine were predictive markers of future T2D risk in our present study, and were also correlated with metabolic markers of insulin resistance (HOMA-IR, HbA_1C_, and TG). The association between BCAAs and new-onset T2D might be explained by following mechanisms. First, the increased BCAAs may evoke catabolic materials (propionyl CoA and succinyl CoA), leading to accumulation of incompletely beta-oxidized fatty acids and glucose, impaired insulin effect, and (ultimately) disturbance of glucose control^32^. Second, the composition and derangement of the intestinal microbiota might induce diabetes development^33^. According to recent research, higher BCAA levels are associated with gastrointestinal microbiome patterns such as *Prevotella copri* and *Bacteroides vulgatus* species^34^, suggesting a role for the human microbiome in the association between BCAAs and T2D development. Our results imply that a healthier dietary pattern significantly reduces the likelihood of future T2D development. Thus, whether certain dietary factors interact with the potential BCAA-biosynthetic component of the gut microbiome pattern, thereby affecting BCAA levels, deserves further investigation. Finally, the mammalian target of rapamycin complex 1 (mTORC1) is important for insulin signaling and secretion and the proliferation of pancreatic beta cells. The primary regulator of mTORC1 signaling is leucine^35^. Long–term elevation of these BCAAs may contribute to the hyperactivation of mTORC1 and Jun N-terminal kinase signaling, presumably causing impaired insulin signaling, and subsequent early dysfunction and destruction of beta cells^36–38^. Despite the accumulating evidence that BCAAs are related to T2D pathogenesis, whether increased BCAA levels are a cause or consequence of T2D has not been elucidated. Very recently, the causal association between BCAA metabolism and T2D was clarified in a Mendelian randomization study, using genetic proxies as instrumental variables^30^. The authors identified the BCAA-raising polymorphisms by a genome-wide approach, and associated them with elevated T2D risk. This result supports a causal pathway of BCAA metabolism in T2D pathogenesis. In the present study, we found that higher levels of alanine and arginine increase the likelihood of incident diabetes. Alanine levels might increase when the metabolism is altered by glutamate turnover^39^, when alanine is released from sites other than skeletal muscle, and when alanine production is disturbed by BCAA catabolism, which is associated with increased T2D risk. Arginine plays multiple beneficial roles against metabolic abnormalities, but might also induce oxidative stress^40^. Collectively, we speculate that altered amino acid metabolisms predict the future risk of T2D through mechanisms involving impaired BCAA metabolism, increased oxidative stress, and increased muscle protein degradation, which precedes the development of T2D.

### Biogenic amines

The biogenic amines spermine and spermidine are involved in several cellular processes, including DNA replication, RNA transcription, and protein biosynthesis^41^. They also trigger glucose-stimulated insulin release^41^; moreover, spermine is a possible glycation inhibitor^42,43^. Consistent with these studies, we found a negative association between blood spermine levels and new-onset T2D, emphasizing the importance of evaluating various metabolites, such as biogenic amines, in new-onset T2D prediction.

### Hexoses

As expected, hexose level was positively associated with new-onset T2D in our present study, even after adjusting for confounding influencers of insulin resistance. Hexose comprises not only glucose, but all six-carbon monosaccharides. Increased hexose level may indicate pancreatic beta-cell dysfunction and insulin resistance. In previous studies, the six-carbon monosaccharide fructose was increased in T2D regardless of glucose level^44^, and higher intake of fructose (including sweetened beverages) was associated with insulin resistance and the risk of new-onset T2D^45^. Our result was consistent with the earlier results^44,45^, confirming that hexose metabolites are valuable for evaluating associations or predicting new-onset T2D in population-based samples.

### Glycerophospholipids and sphingomyelin

Consistent with earlier studies^6,7^, we found a possible involvement of phospholipid metabolism in incident T2D among our Korean population. Specifically, two LysoPC metabolites (17:0 and 18:2), 8 PC aa metabolites (32:1, 34:1, 36:1, 38:0, 40:1, 40:5, 42:1, and 42:5), 2 PC ae (34:3 and 36:3), SM(OH) 22:2, and SM 16:1 were significant predictors of T2D risk in this prospective study. Phospholipids such as PC and SM are the dominant components of cellular membranes and play an important role in cellular signal transduction^46^. They also constitute most of the human lipoproteins^47^. The LysoPC 17:0 and 18:2 were negatively associated with incident T2D in our cohort. LysoPC a 17:0, found exclusively in dairy foods, is particularly effective in reducing T2D risk^48^, indicating the beneficial effect of regularly consuming dairy products. LysoPC is a signaling molecule involved in atherogenic and inflammatory processes^49^, of which the major function needs to be clearly elucidated. PC aa is essential for the hepatic release of TG-rich very-low-density lipoprotein^46^. On the other hand, PC ae is an antioxidant that inhibits lipoprotein oxidation^50^. Previous studies have shown that PC ae levels are reduced in obese or insulin-resistant subjects^50,51^. Furthermore, reduced SM synthesis is associated with increased levels of reactive oxygen species and reduced insulin secretion^52^. The significant contributions of LysoPC, PC, and SM to T2D development in the present study, and the correlations of these metabolites with blood phenotype (observed as expected), support the hypothesis that altered choline-containing phospholipid metabolism potentiates the development of T2D.

Several recent studies have also identified the metabolites associated with food intake, and related them to the metabolic diseases at the population level^53–58^. Untargeted metabolomics analysis in the subset of African Americans from the Atherosclerosis Risk in Communities (ARIC) Study cohort identified 48 pairs of significant association between diet and metabolites^53^. Specifically, ‘sugar-rich foods and beverages’ were associated with metabolites related to oxidative stress and lipid profiles^53^. Another approach to reveal the association between dietary pattern and plasma/urinary metabolites reported that O-acetylcarnitine and phenylacetylglutamine are positively associated with different food groups, red-meat and vegetable intakes, respectively^54^. Given that diet can be a primary and secondary (by induction of metabolic responses) source of metabolites^57,58^, we further tested the interrelationships among dietary quality, metabolites, and T2D incidence. In this study, only 22 metabolites were significant predictors of incident T2D. After controlling for covariates, multiple regression analysis revealed that a healthier diet was significantly associated with reduced PC aa 32:1 level and increased the SM(OH) 22:2 level. To test whether each of these two metabolites may act as a mediator in the observed negative association between dietary quality and T2D risk, both dietary score and metabolite were considered in the Cox proportional regression model. The result showed that each metabolite and dietary score remained significant in combination, although the HRs were somewhat attenuated. This indicates that a better diet quality, characterized by well-balanced nutrient intakes reduces the future T2D risk both dependently and independently of intermediate metabolites. Given that food intake and relevant nutrients possibly interact with the environment and the gut microbiome, and that these interactions would be reflected in the blood metabolites, the links among dietary intake, gut microbiota and metabolic biomarkers and their associations with T2D must be further investigated. Indeed, the consumption of PC-containing foods such as meat and eggs reportedly increases the T2D risk^59^, possibly through bacterial transformation of PC and consequent production of trimethylamine N-oxide^60,61^. In addition to the complexity and differences of etiology of T2D and environmental factors including diet, ethnic difference also should be considered to interpret metabolic profile in a specific population. Because the response to the specific stimulus (e.g. chemicals, nutrients, etc.) can be distinct depending on gender and ethnic group^62^, separate analysis in a certain population is required to apply the identified metabolites for planning the preventive strategy as well as for pharmacological targeting to treat T2D.

In conclusion, our study showed that metabolites relevant to amino acids, biogenic amines, spermine, hexoses, and choline-containing phospholipid metabolism are associated with incident T2D in a prospective community-based cohort study. Through targeted metabolite profiling, we elucidated the underlying biochemical pathway of T2D pathogenesis. However, many of the covered metabolites were choline-containing phospholipids with structural similarities and metabolic interrelationships, which hindered the comprehensive assessment of the metabolic alterations preceding T2D. Our results confirm that metabolites beyond the conventional T2D risk factors are important for preventing or controlling the development of new-onset T2D. With this knowledge, we can develop strategies for early intervention against average-risk T2D.

## Electronic supplementary material

Supplementary tables
