# Supplementary material for: Serum metabolite profile associated with incident type 2 diabetes in Koreans: findings from the Korean Genome and Epidemiology Study
Source: Sci Rep. 2018 May 29;8:8207. doi: 10.1038/s41598-018-26320-9 (PMC5974077; doi:10.1038/s41598-018-26320-9)
Supplement: Supplementary file 1 — Supplementary tables [file 41598_2018_26320_MOESM1_ESM.docx]

**Serum metabolite profile associated with incident type 2 diabetes in Koreans: findings from the Korean Genome and Epidemiology Study**

Soo Jin Yang^1^, So-Young Kwak^2^, Garam Jo^2^, Tae-Jin Song^3^, Min-Jeong Shin^2,*^

^1^Department of Food and Nutrition, Seoul Women's University, Seoul 01797, Republic of Korea, ^2^Department of Public Health Sciences, BK21PLUS Program in Embodiment: Health-Society Interaction, Graduate School, Korea University, Seoul 02841, Republic of Korea, ^3^Department of Neurology, Ewha Womans University School of Medicine, Seoul 07985, Republic of Korea

**Supplementary Table 1. Nutrient intakes and biomarkers according to quartiles of modified RFS**

|  | Total  (n=1939) | Recommended Food Score (RFS) | | | | p-value^†^ |
| --- | --- | --- | --- | --- | --- | --- |
|  |  | Q1 (n=465) | Q2 (n=486) | Q3 (n=480) | Q4 (n=508) |  |
| Score, mean/median (range) | 19.6/20 (0-48) | 7.6/8 (0-12) | 16.1/16 (13-19) | 22.5/22 (20-25) | 31.2/30 (26-48) | - |
| Energy intake, kcal | 1775.2 ± 13.6 | 1449.5 ± 19.6^a^ | 1665.2 ± 22.2^b^ | 1825.7 ± 22.8^c^ | 2127.0 ± 31.5^d^ | <0.001 |
| Nutrient intake |  |  |  |  |  |  |
| % Carbohydrate | 73.9 ± 0.3 | 78.7 ± 0.3^a^ | 75.0 ± 0.3^b^ | 72.9 ± 0.3^c^ | 69.4 ± 0.3^d^ | <0.001 |
| % Protein | 13.0 ± 0.1 | 11.4 ± 0.1^a^ | 12.7 ± 0.1^b^ | 13.1 ± 0.1^c^ | 14.5 ± 0.1^d^ | <0.001 |
| % Fat | 13.2 ± 0.1 | 9.9 ± 0.2^a^ | 12.3 ± 0.3^b^ | 14.0 ± 0.2^c^ | 16.1 ± 0.2^d^ | <0.001 |
| Calcium^1^, mg | 234.2 ± 2.4 | 167.1 ± 3.9^a^ | 230.0 ± 4.8^b^ | 241.6 ± 4.3^b^ | 291.9 ± 4.6^c^ | <0.001 |
| Phosphorus^1^, mg | 493.2 ± 2.1 | 427.9 ± 3.5^a^ | 486.9 ± 4.0^b^ | 501.1 ± 3.6^b^ | 551.3 ± 4.0^c^ | <0.001 |
| Iron^1^, mg | 5.35 ± 0.04 | 4.31 ± 0.06^a^ | 5.17 ± 0.06^b^ | 5.50 ± 0.06^c^ | 6.33 ± 0.07^d^ | <0.001 |
| Potassium^1^, mg | 1252.2 ± 9.2 | 972.4 ± 18.0^a^ | 1209.5 ± 14.3^b^ | 1287.4 ± 14.8^c^ | 1512.6 ± 17.3^d^ | <0.001 |
| Sodium^1^, mg | 1561.9 ± 18.4 | 1437.5 ± 43.6^a^ | 1649.2 ± 40.0^b^ | 1536.1 ± 31.0^ab^ | 1615.3 ± 31.7^b^ | <0.001 |
| Zinc^1^, ug | 4.25 ± 0.02 | 3.83 ± 0.03^a^ | 4.16 ± 0.03^b^ | 4.32 ± 0.06^b^ | 4.67 ± 0.04^c^ | <0.001 |
| Vitamin A^1^, R.E. | 261.7 ± 3.8 | 188.7 ± 7.1^a^ | 263.0 ± 7.8^b^ | 260.9 ± 6.1^b^ | 327.3 ± 7.5^c^ | <0.001 |
| Vitamin B1^1^, mg | 0.560 ± 0.003 | 0.483 ± 0.005^a^ | 0.561 ± 0.006^b^ | 0.567 ± 0.005^b^ | 0.621 ± 0.005^c^ | <0.001 |
| Vitamin B2^1^, mg | 0.480 ± 0.004 | 0.367 ± 0.006^a^ | 0.458 ± 0.006^b^ | 0.495 ± 0.006^c^ | 0.588 ± 0.006^d^ | <0.001 |
| Niacin^1^, mg | 7.66 ± 0.04 | 6.65 ± 0.07^a^ | 7.49 ± 0.07^b^ | 7.79 ± 0.07^c^ | 8.62 ± 0.07^d^ | <0.001 |
| Vitamin C^1^, mg | 56.4 ± 0.7 | 37.3 ± 1.1^a^ | 53.7 ± 1.2^b^ | 58.3 ± 1.1^c^ | 74.6 ± 1.3^d^ | <0.001 |
| Vitamin B6^1^, mg | 0.878 ± 0.005 | 0.748 ± 0.009^a^ | 0.873 ± 0.009^b^ | 0.888 ± 0.008^b^ | 0.991 ± 0.09^c^ | <0.001 |
| Folate^1^, ug | 121.9 ± 1.2 | 97.8 ± 2.5^a^ | 122.2 ± 2.4^b^ | 120.9 ± 2.0^b^ | 144.4 ± 2.3^c^ | <0.001 |
| Retinol^1^, ug | 29.8 ± 0.6 | 18.3 ± 1.0^a^ | 26.2 ± 1.2^b^ | 32.3 ± 1.0^c^ | 41.3 ± 1.0^d^ | <0.001 |
| Carotene^1^, ug | 1349.2 ± 21.7 | 990.7 ± 41.4^a^ | 1381.4 ± 45.9^b^ | 1327.0 ± 35.6^b^ | 1663.2 ± 44.3^c^ | <0.001 |
| Fiber^1^, g | 3.35 ± 0.03 | 2.83 ± 0.06^a^ | 3.37 ± 0.06^b^ | 3.39 ± 0.05^b^ | 3.76 ± 0.05^c^ | <0.001 |
| Vitamin E^1^, mg | 4.33 ± 0.03 | 3.40 ± 0.07^a^ | 4.02 ± 0.05^b^ | 4.45 ± 0.05^c^ | 5.35 ± 0.07^d^ | <0.001 |
| Cholesterol^1^, mg | 75.2 ± 1.2 | 47.8 ± 2.4^a^ | 67.2 ± 2.2^b^ | 82.5 ± 2.1^c^ | 100.7 ± 2.0^d^ | <0.001 |

Values are expressed as means ± standard error for continuous variables and percentages and numbers counts for categorical variables ^†^Statistical differences were determined using chi square test for categorical variables and one-way analysis of variance (ANOVA) for continuous variables with Bonferroni’s multiple correction (p < 0.05) ^1^ Micronutrients intake was calculated as value per 1,000 calories

**Supplementary Table 2. Risk of Type 2 Diabetes Mellitus according to metabolites**

| Metabolites | HRs | p value**^†^** | q value**^‡^** |
| --- | --- | --- | --- |
| Acylcarnitine |  |  |  |
| Carnitine | 0.918 | 0.174 | 0.953 |
| Acetylcarnitine | 0.958 | 0.510 | 0.953 |
| Propionylcarinitine | 1.078 | 0.248 | 0.953 |
| Butyrylcarnitine | 0.958 | 0.497 | 0.953 |
| Valerylcarnitine | 1.046 | 0.490 | 0.953 |
| Pimelylcarnitine | 0.892 | 0.080 | 0.953 |
| Octanoylcarnitine | 0.915 | 0.163 | 0.953 |
| Tetradecenoylcarnitine | 0.909 | 0.146 | 0.953 |
| Tetradecadienylcarnitine | 1.016 | 0.797 | 0.953 |
| Hexadecanoylcarnitine | 0.950 | 0.423 | 0.953 |
| Ocatdecanoylcarnitine | 0.952 | 0.450 | 0.953 |
| Octadecenoylcarnitine | 1.098 | 0.110 | 0.953 |
| Octadecadienylcarnitine | 0.964 | 0.582 | 0.953 |
| Amino acids |  |  |  |
| Alanine | 1.470 | <0.001^*^ | <0.001^*^ |
| Arginine | 1.274 | <0.001^*^ | 0.032^*^ |
| Asparagine | 0.900 | 0.092 | 0.953 |
| Aspartate | 1.045 | 0.466 | 0.953 |
| Citrulline | 1.041 | 0.537 | 0.953 |
| Glutamine | 1.039 | 0.548 | 0.953 |
| Glutamate | 1.177 | 0.006^*^ | 0.560 |
| Glycine | 0.808 | 0.002^*^ | 0.180 |
| Histidine | 1.072 | 0.267 | 0.953 |
| Isoleucine | 1.354 | <0.001^*^ | 0.001^*^ |
| Leucine | 1.227 | 0.002^*^ | 0.206 |
| Lysine | 1.118 | 0.074 | 0.953 |
| Methionine | 1.177 | 0.013^*^ | 0.953 |
| Ornithine | 1.044 | 0.495 | 0.953 |
| Phenylalanine | 1.223 | 0.002^*^ | 0.190 |
| Proline | 1.262 | <0.001^*^ | 0.022^*^ |
| Serine | 0.975 | 0.684 | 0.953 |
| Threonine | 0.989 | 0.869 | 0.953 |
| Tryptophan | 1.082 | 0.218 | 0.953 |
| Tyrosine | 1.272 | <0.001^*^ | 0.027^*^ |
| Valine | 1.456 | <0.001^*^ | <0.001^*^ |
| Biogenic amines |  |  |  |
| Acetylornithine | 0.897 | 0.056 | 0.953 |
| Asymmetric dimethylarginine | 1.035 | 0.586 | 0.953 |
| Creatinine | 0.871 | 0.075 | 0.953 |
| Kynurenine | 0.966 | 0.585 | 0.953 |
| Putrescine | 0.978 | 0.704 | 0.953 |
| Sarcosine | 0.992 | 0.906 | 0.953 |
| Serotonin | 1.043 | 0.495 | 0.953 |
| Spermidine | 0.947 | 0.350 | 0.953 |
| Spermine | 0.734 | <0.001^*^ | <0.001^*^ |
| Taurine | 1.015 | 0.813 | 0.953 |
| Sugar |  |  |  |
| Hexose | 1.757 | <0.001^*^ | <0.001^*^ |
| Lyso Phosphatidylcholine |  |  |  |
| Lyso Phosphatidylcholine acyl C16:0 | 1.007 | 0.917 | 0.953 |
| Lyso Phosphatidylcholine acyl C16:1 | 1.038 | 0.563 | 0.953 |
| Lyso Phosphatidylcholine acyl C17:0 | 0.791 | <0.001^*^ | 0.014^*^ |
| Lyso Phosphatidylcholine acyl C18:0 | 0.925 | 0.224 | 0.953 |
| Lyso Phosphatidylcholine acyl C18:1 | 0.809 | 0.002^*^ | 0.220 |
| Lyso Phosphatidylcholine acyl C18:2 | 0.746 | <0.001^*^ | 0.001^*^ |
| Lyso Phosphatidylcholine acyl C20:3 | 1.020 | 0.753 | 0.953 |
| Lyso Phosphatidylcholine acyl C20:4 | 0.941 | 0.354 | 0.953 |
| Phosphatidylcholine diacyl |  |  |  |
| Phosphatidylcholine diacyl C28:1 | 1.051 | 0.450 | 0.953 |
| Phosphatidylcholine diacyl C30:0 | 1.211 | 0.002^*^ | 0.206 |
| Phosphatidylcholine diacyl C32:0 | 1.222 | 0.001^*^ | 0.099 |
| Phosphatidylcholine diacyl C32:1 | 1.417 | <0.001^*^ | <0.001^*^ |
| Phosphatidylcholine diacyl C32:3 | 0.927 | 0.232 | 0.953 |
| Phosphatidylcholine diacyl C34:1 | 1.317 | <0.001^*^ | 0.002^*^ |
| Phosphatidylcholine diacyl C34:2 | 1.160 | 0.014^*^ | 0.953 |
| Phosphatidylcholine diacyl C34:3 | 1.075 | 0.246 | 0.953 |
| Phosphatidylcholine diacyl C34:4 | 1.170 | 0.013^*^ | 0.953 |
| Phosphatidylcholine diacyl C36:0 | 0.817 | 0.001^*^ | 0.139 |
| Phosphatidylcholine diacyl C36:1 | 1.257 | <0.001^*^ | 0.030^*^ |
| Phosphatidylcholine diacyl C36:2 | 1.114 | 0.073 | 0.953 |
| Phosphatidylcholine diacyl C36:3 | 1.085 | 0.183 | 0.953 |
| Phosphatidylcholine diacyl C36:4 | 1.139 | 0.042^*^ | 0.953 |
| Phosphatidylcholine diacyl C36:5 | 1.207 | 0.002^*^ | 0.214 |
| Phosphatidylcholine diacyl C36:6 | 1.082 | 0.211 | 0.953 |
| Phosphatidylcholine diacyl C38:0 | 0.799 | <0.001^*^ | 0.045^*^ |
| Phosphatidylcholine diacyl C38:1 | 0.831 | 0.002^*^ | 0.247 |
| Phosphatidylcholine diacyl C38:3 | 1.152 | 0.026^*^ | 0.953 |
| Phosphatidylcholine diacyl C38:4 | 1.078 | 0.236 | 0.953 |
| Phosphatidylcholine diacyl C38:5 | 1.215 | 0.002^*^ | 0.171 |
| Phosphatidylcholine diacyl C38:6 | 1.150 | 0.027^*^ | 0.953 |
| Phosphatidylcholine diacyl C40:1 | 0.791 | <0.001^*^ | 0.034^*^ |
| Phosphatidylcholine diacyl C40:2 | 0.892 | 0.061 | 0.953 |
| Phosphatidylcholine diacyl C40:3 | 0.893 | 0.060 | 0.953 |
| Phosphatidylcholine diacyl C40:4 | 1.037 | 0.564 | 0.953 |
| Phosphatidylcholine diacyl C40:5 | 1.330 | <0.001^*^ | 0.001^*^ |
| Phosphatidylcholine diacyl C40:6 | 1.211 | 0.002^*^ | 0.214 |
| Phosphatidylcholine diacyl C42:0 | 0.857 | 0.015^*^ | 0.953 |
| Phosphatidylcholine diacyl C42:1 | 0.797 | <0.001^*^ | 0.037^*^ |
| Phosphatidylcholine diacyl C42:2 | 0.865 | 0.023 | 0.953 |
| Phosphatidylcholine diacyl C42:4 | 0.930 | 0.223 | 0.953 |
| Phosphatidylcholine diacyl C42:5 | 1.239 | <0.001^*^ | 0.027^*^ |
| Phosphatidylcholine diacyl C42:6 | 1.049 | 0.413 | 0.953 |
| Phosphatidylcholine acyl-alkyl |  |  |  |
| Phosphatidylcholine acyl-alkyl C30:0 | 0.919 | 0.183 | 0.953 |
| Phosphatidylcholine acyl-alkyl C32:1 | 1.004 | 0.953 | 0.953 |
| Phosphatidylcholine acyl-alkyl C32:2 | 0.913 | 0.134 | 0.953 |
| Phosphatidylcholine acyl-alkyl C34:0 | 1.007 | 0.913 | 0.953 |
| Phosphatidylcholine acyl-alkyl C34:1 | 1.064 | 0.313 | 0.953 |
| Phosphatidylcholine acyl-alkyl C34:2 | 0.857 | 0.013^*^ | 0.953 |
| Phosphatidylcholine acyl-alkyl C34:3 | 0.773 | <0.001^*^ | 0.004^*^ |
| Phosphatidylcholine acyl-alkyl C36:0 | 0.909 | 0.143 | 0.953 |
| Phosphatidylcholine acyl-alkyl C36:1 | 1.085 | 0.184 | 0.953 |
| Phosphatidylcholine acyl-alkyl C36:2 | 0.902 | 0.091 | 0.953 |
| Phosphatidylcholine acyl-alkyl C36:3 | 0.796 | <0.001^*^ | 0.034^*^ |
| Phosphatidylcholine acyl-alkyl C36:4 | 0.877 | 0.036^*^ | 0.953 |
| Phosphatidylcholine acyl-alkyl C36:5 | 0.909 | 0.131 | 0.953 |
| Phosphatidylcholine acyl-alkyl C38:0 | 0.949 | 0.411 | 0.953 |
| Phosphatidylcholine acyl-alkyl C38:1 | 0.890 | 0.036^*^ | 0.953 |
| Phosphatidylcholine acyl-alkyl C38:2 | 1.070 | 0.260 | 0.953 |
| Phosphatidylcholine acyl-alkyl C38:3 | 0.894 | 0.069 | 0.953 |
| Phosphatidylcholine acyl-alkyl C38:4 | 0.879 | 0.036^*^ | 0.953 |
| Phosphatidylcholine acyl-alkyl C38:5 | 0.936 | 0.284 | 0.953 |
| Phosphatidylcholine acyl-alkyl C38:6 | 0.897 | 0.088 | 0.953 |
| Phosphatidylcholine acyl-alkyl C40:1 | 0.810 | 0.001^*^ | 0.075 |
| Phosphatidylcholine acyl-alkyl C40:2 | 0.862 | 0.016^*^ | 0.953 |
| Phosphatidylcholine acyl-alkyl C40:3 | 0.817 | 0.001^*^ | 0.107 |
| Phosphatidylcholine acyl-alkyl C40:4 | 0.807 | 0.001^*^ | 0.068 |
| Phosphatidylcholine acyl-alkyl C40:5 | 0.988 | 0.841 | 0.953 |
| Phosphatidylcholine acyl-alkyl C40:6 | 0.870 | 0.028^*^ | 0.953 |
| Phosphatidylcholine acyl-alkyl C42:0 | 0.860 | 0.018^*^ | 0.953 |
| Phosphatidylcholine acyl-alkyl C42:1 | 0.799 | 0.001^*^ | 0.062 |
| Phosphatidylcholine acyl-alkyl C42:2 | 0.877 | 0.030^*^ | 0.953 |
| Phosphatidylcholine acyl-alkyl C42:3 | 0.844 | 0.007^*^ | 0.658 |
| Phosphatidylcholine acyl-alkyl C42:4 | 0.811 | 0.001^*^ | 0.106 |
| Phosphatidylcholine acyl-alkyl C42:5 | 0.896 | 0.069 | 0.953 |
| Phosphatidylcholine acyl-alkyl C44:3 | 0.886 | 0.042^*^ | 0.953 |
| Phosphatidylcholine acyl-alkyl C44:4 | 0.879 | 0.036^*^ | 0.953 |
| Phosphatidylcholine acyl-alkyl C44:5 | 0.944 | 0.339 | 0.953 |
| Phosphatidylcholine acyl-alkyl C44:6 | 0.853 | 0.012^*^ | 0.953 |
| Sphingolipids |  |  |  |
| Hydroxysphingomyelin C14:1 | 0.807 | 0.001^*^ | 0.121 |
| Hydroxysphingomyelin C16:1 | 0.850 | 0.011^*^ | 0.953 |
| Hydroxysphingomyelin C22:1 | 0.971 | 0.655 | 0.953 |
| Hydroxysphingomyelin C22:2 | 0.719 | <0.001^*^ | <0.001^*^ |
| Hydroxysphingomyelin C24:1 | 0.921 | 0.181 | 0.953 |
| Sphingomyelin C16:0 | 0.849 | 0.008^*^ | 0.802 |
| Sphingomyelin C16:1 | 0.790 | <0.001^*^ | 0.043^*^ |
| Sphingomyelin C18:0 | 1.035 | 0.578 | 0.953 |
| Sphingomyelin C18:1 | 0.851 | 0.015^*^ | 0.953 |
| Sphingomyelin C24:0 | 1.019 | 0.757 | 0.953 |
| Sphingomyelin C24:1 | 0.832 | 0.003^*^ | 0.259 |
| Sphingomyelin C26:1 | 0.836 | 0.003^*^ | 0.314 |

Hazard Ratios (HRs) were obtained with cox proportional hazards regression model adjusting for sex, age, energy intake, body mass index, metabolic equivalent, smoking status, drinking status, household income, education level, consumption of coffee, red meat, and whole grain, and history of hypertension. † The values of metabolites used in this analysis were log-transformed and normalized using z score. ‡ False discovery rate corrected p-value defined by Benjamini-Hochberg method (q <0.05)

**Supplementary table 3. Differences in the levels of 22 selected metabolites related to Type 2 Diabetes risk based on lowest quartile versus highest quartile of modified RFS**

| Metabolites | Recommended Food Score (RFS) | | Beta^*^ | p value**^†^** | q value**^‡^** |
| --- | --- | --- | --- | --- | --- |
|  | Q1  (n=465) | Q4  (n=508) |  |  |  |
| Alanine | 498.213 ± 4.903 | 485.226 ± 4.789 | -0.05 | 0.542 | 0.958 |
| Arginine | 124.834 ± 1.618 | 126.271 ± 1.622 | -0.01 | 0.958 | 0.958 |
| Isoleucine | 82.307 ± 0.918 | 81.513 ± 0.821 | -0.16 | 0.050 | 0.605 |
| Proline | 168.183 ± 2.399 | 160.555 ± 2.028 | -0.22 | 0.011 | 0.160 |
| Tyrosine | 71.802 ± 0.700 | 71.148 ± 0.636 | -0.06 | 0.519 | 0.958 |
| Valine | 213.523 ± 1.786 | 218.886 ± 1.761 | 0.04 | 0.656 | 0.958 |
| Spermine | 0.213 ± 0.003 | 0.210 ± 0.002 | -0.16 | 0.067 | 0.681 |
| Hexose | 4935.688 ± 37.519 | 4783.740 ± 33.240 | -0.18 | 0.035 | 0.452 |
| Lyso Phosphatidylcholine acyl C17:0 | 3.542 ± 0.044 | 3.728 ± 0.047 | 0.16 | 0.072 | 0.681 |
| Lyso Phosphatidylcholine acyl C18:2 | 31.174 ± 0.469 | 31.540 ± 0.425 | 0.03 | 0.682 | 0.958 |
| Phosphatidylcholine diacyl C32:1 | 14.487 ± 0.381 | 11.128 ± 0.246 | -0.28 | 0.001 | 0.014 |
| Phosphatidylcholine diacyl C34:1 | 178.614 ± 2.773 | 157.065 ± 2.000 | -0.26 | 0.004 | 0.076 |
| Phosphatidylcholine diacyl C36:1 | 43.986 ± 0.721 | 38.607 ± 0.495 | -0.25 | 0.003 | 0.081 |
| Phosphatidylcholine diacyl C38:0 | 4.181 ± 0.057 | 4.813 ± 0.057 | 0.23 | 0.006 | 0.097 |
| Phosphatidylcholine diacyl C40:1 | 0.535 ± 0.007 | 0.600 ± 0.008 | 0.20 | 0.020 | 0.279 |
| Phosphatidylcholine diacyl C40:5 | 16.666 ± 0.324 | 14.170 ± 0.225 | -0.24 | 0.004 | 0.076 |
| Phosphatidylcholine diacyl C42:1 | 0.484± 0.006 | 0.538 ± 0.007 | 0.24 | 0.006 | 0.095 |
| Phosphatidylcholine diacyl C42:5 | 0.566 ± 0.010 | 0.526 ± 0.008 | -0.15 | 0.076 | 0.681 |
| Phosphatidylcholine acyl-alkyl C34:3 | 6.654 ± 0.092 | 7.140 ± 0.093 | 0.12 | 0.161 | 0.958 |
| Phosphatidylcholine acyl-alkyl C36:3 | 5.964 ± 0.072 | 5.620 ± 0.071 | 0.07 | 0.392 | 0.958 |
| Hydroxysphingomyelin C22:2 | 8.670 ± 0.105 | 9.604 ± 0.106 | 0.26 | 0.001 | 0.011 |
| Sphingomyelin C16:1 | 17.036 ± 0.173 | 17.356 ± 0.154 | 0.13 | 0.103 | 0.823 |

^*^ Beta coefficient was using multiple linear regression model adjusting for age, sex, energy intake, metabolic equivalent, smoking status, drinking status, household income and education level. **†** The values of metabolites used in this analysis were log-transformed and normalized using z score. **‡** False discovery rate corrected p-value defined by Benjamini-Hochberg method (q < 0.05)
